# Negative Effects of Cyanotoxins and Adaptative Responses of *Daphnia*

**DOI:** 10.3390/toxins14110770

**Published:** 2022-11-07

**Authors:** Anke Schwarzenberger

**Affiliations:** Limnological Institute, University Konstanz, Mainaustr. 252, 78464 Konstanz, Germany; anke.schwarzenberger@uni-konstanz.de

**Keywords:** carboxypeptidase inhibitors, protease inhibitors, microcystins, anatoxin, cylindrosper, mopsin, anthropogenic stressors

## Abstract

The plethora of cyanobacterial toxins are an enormous threat to whole ecosystems and humans. Due to eutrophication and increases in lake temperatures from global warming, changes in the distribution of cyanobacterial toxins and selection of few highly toxic species/strains are likely. Globally, one of the most important grazers that controls cyanobacterial blooms is *Daphnia*, a freshwater model organism in ecology and (eco)toxicology. *Daphnia*–cyanobacteria interactions have been studied extensively, often focusing on the interference of filamentous cyanobacteria with *Daphnia*’s filtering apparatus, or on different nutritional constraints (the lack of essential amino acids or lipids) and grazer toxicity. For a long time, this toxicity only referred to microcystins. Currently, the focus shifts toward other deleterious cyanotoxins. Still, less than 10% of the total scientific output deals with cyanotoxins that are not microcystins; although these other cyanotoxins can occur just as frequently and at similar concentrations as microcystins in surface water. This review discusses the effects of different cyanobacterial toxins (hepatotoxins, digestive inhibitors, neurotoxins, and cytotoxins) on *Daphnia* and provides an elaborate and up-to-date overview of specific responses and adaptations of *Daphnia*. Furthermore, scenarios of what we can expect for the future of *Daphnia*–cyanobacteria interactions are described by comprising anthropogenic threats that might further increase toxin stress in *Daphnia*.

## 1. Introduction

“*We must go deeper into greater pain; for it is not permitted that we stay*”

from “The Divine Comedy” by Dante Alighieri

Both in terrestrial (e.g., [1]) and aquatic ecosystems (e.g., [2]), herbivore–plant interactions play an important role in the trophic transfer of energy (in the form of carbon) from primary producers to consumers. In lakes, *Cladocera*—especially the aquatic keystone genus *Daphnia*—are the most important consumers of planktonic algae. *Daphnia* cannot select food particles based on their dietary quality [3], and therefore ingest all particles that are in the range of the filter-feeding appendices of their thoracopods [4,5]. Cyanobacteria are an especially poor food source for *Daphnia* [6]. They negatively affect somatic growth rates [7,8] and food uptake [9,10]. Furthermore, declines in the biomass of *Daphnia* that are caused by cyanobacteria have been observed in several field studies [11,12,13,14].

A variety of characteristics of cyanobacteria are responsible for the depression of *Daphnia*: mechanical interference of filamentous forms with the filtering apparatuses of *Daphnia* [15], the production of feeding deterrents [16], the lack of certain amino acids [17,18], polyunsaturated fatty acids and sterols [19,20,21] and, last but not least, the production of toxins causing the death of *Daphnia* (e.g., [22]).

Cyanobacteria produce an extraordinarily high number of very diverse toxins [23,24]. According to their mode of action, these cyanobacterial toxins have been divided into hepatotoxins, cytotoxins, digestive inhibitors, neurotoxins, dermatotoxins and irritants (e.g., [25]). Cyanobacteria can occur in very high densities. Such cyanobacterial blooms are seldom monoclonal but often consist of several strains, species and/or genera simultaneously (e.g., [26,27]), and a succession of cyanobacterial species over a season or subsequent years had been observed [28,29,30,31,32]. Furthermore, the separate species co-occurring in a bloom often produce multiple classes of toxins [33,34,35,36], so cyanobacterial blooms often contain a mix of different toxin types (e.g., [26,27]).

Cyanobacterial blooms have massively increased in frequency because of global warming and the eutrophication of freshwater systems [37] and are an increasing threat to livestock, pets and human health [38]. Furthermore, a recent study by Mantzouki et al. [39] found that more than eutrophication, the direct and indirect effects of temperature were the main drivers of the spatial distribution of different cyanotoxins (e.g., microcystins, anatoxin and cylindrospermopsin) and their concentrations in European freshwater systems. This study also found that high water stability and high temperatures reduced the variability of toxin types in lakes and showed that temperature-related mechanisms led to the selective development of well-adapted cyanobacterial strains, which potentially promotes the dominance of a few highly toxic strains in the future.

Different types of cyanotoxins affect *Daphnia* in different ways and target different enzymes and/or receptors. However, *Daphnia* are no helpless victims to cyanotoxins and show different general and also many specific responses in order to substitute or increase inhibited molecules and to export and detoxify cyanotoxins (Table 1). Furthermore, *Daphnia* populations that have come into contact with cyanotoxins over long periods are often locally adapted [40,41]. Below are the effects of the cytotoxin cylindrospermopsin, digestive inhibitors (i.e., protease and caboxypeptidase inhibitors), the hepatotoxic microcystins and the neurotoxin anatoxin-a on *Daphnia* and the respective responses and adaptations of *Daphnia*.

## 2. Cylindrospermopsin (CYN)

The cytotoxin CYN occurs globally and is produced by different cyanobacterial species [42]. Although CYN was detected less frequently than other toxin types in Europe [39], it has been shown to be present over a very long time in aquatic systems due to the succession of different CYN-producing bloom species [29]. Therefore, Mantzouki et al. [39] suggested that this indicates that CYN producers can exclude cyanobacteria that produce other toxin types. In vertebrates, CYN affects different metabolic pathways [43,44], suppresses the immune system [45] and leads to oxidative stress [46], genotoxicity [47,48] and abnormal function of hepatocytes [49].

Not much is known about the effects on and the responses of *Daphnia* to this cytotoxin. The dissociation of gut epithelial cells of *D. magna* could not clearly be attributed to CYN [50]. However, Nogueira et al. [51] showed that *D. magna* incorporates CYN in its tissue, and feeding a CYN-producing *Cylindrospermopsis raciborskii* led to higher mortality and less growth compared to feeding a *C. raciborskii* strain without CYN. Furthermore, when *D. magna* was exposed to CYN for 24 h, a tendency for increased glutathione-S transferase (GST) enzyme activities was observed.

Since CYN has been associated with the killing of humans in the past [42] and will probably occur more frequently with increasing temperatures, the responses of *Daphnia*—which might have the potential to control CYN-producing blooms—need to be investigated more thoroughly in the future.

## 3. Inhibitors of Protein Cleaving Enzymes

### 3.1. Protein Digestion of Daphnia

The first description of *Daphnia*’s digestive enzymes was reported by Hasler in 1935 [52]. Since then, the activity of *Daphnia*’s protein-cleaving digestive enzymes, e.g., proteases and carboxypeptidases, has been investigated in only a few other studies [53,54,55]. Protein digestion takes place in *Daphnia*’s midgut, and the respective enzymes for protein digestion are released from the midgut gland. Those enzymes include protein phosphatases, carboxypeptidases and serine proteases. The production of protein-cleaving enzymes is probably controlled by *Daphnia*’s circadian clock since they show a rhythmic gene expression pattern over 24 h [56]. Interestingly, the enzyme activity is at a constant level over 24 h and does not follow its gene expression peak.

In *Daphnia*, serine proteases represent the most important digestive enzymes [57]. The two types of serine proteases that have been identified in *Daphnia*’s gut are trypsins and chymotrypsins [54]. These two types of serine proteases are differentially affected by temperature. At high temperatures, the gene expression of trypsins is increased, which is the case for chymotrypsins at low temperatures [58]. Trypsins and chymotrypsins show a huge gene family expansion in *D. pulex* (> 250 protease genes [59]) indicating that *Daphnia* is capable of modulating the digestive enzymes depending on the available food items. Accordingly, it has been shown that within 24 h, *D. magna* can shift to another protease isoform pattern when food quality is low [60]. Furthermore, *D. pulex* increases protease and phosphatase gene expression when grown on a nontoxic cyanobacterium [55]). This cost-effective overall increase of gene expression and activity probably adds to the frequently observed low biomass and energy transfer efficiency from cyanobacteria to *Daphnia* [20].

### 3.2. Carboxypeptidase (CXP) Inhibitors: Anabaenopeptins

Anabaenopeptins are a group of harmful secondary metabolites produced by different cyanobacterial species (e.g., [61]) that can act as CXP inhibitors [62]. Anabaenopeptins occur frequently in surface waters as microcystins (reviewed in [39,63]) and occur in a multitude of structural isoforms [64].

Methanolic extracts from different *Planktothrix* strains that produce anabaenopeptins [35] have been shown to inhibit *D. magna*’s CXP activity [65]. Furthermore, it has recently been shown that *D. magna* respond to the presence of CXP inhibitors in their food by increasing CXP gene expression [65]. It is not known whether this increased gene expression is an adaptive response to CXP inhibitors, but a deeper investigation is urgently needed as anabaenopeptin-producing cyanobacteria increase in frequency worldwide [63].

### 3.3. Protease Inhibitors (PIs)

PIs belong to the best-studied cyanotoxins regarding *Daphnia*–cyanobacteria interactions. Over the recent decades, potent serine PIs that mostly belong to Ahp-cyclodepsipeptides [66] have frequently been detected in (nearly every) cyanobacterial bloom (e.g., [30,39,67,68,69,70,71]). Different PIs are produced by different cyanobacterial genera [72,73,74], and single strains of the same cyanobacterial species often produce more than one type of PI [33,34,36,65,75,76].

*Daphnia* are strongly affected by PIs; e.g., when ingesting food particles that contain PIs, *Daphnia* show feeding inhibition and reduced somatic growth rates [8,77]. Furthermore, PIs have been linked to an observed mass mortality of *Daphnia* in a Swiss lake during a *Planktothrix* bloom [14]. On a molecular level, PIs have been shown to inhibit digestive serine proteases of *Daphnia* either in vitro or in situ [54,60,65,70,77]. The production of cyanobacterial PIs is dependent on nutrient availability. Schwarzenberger et al. [34] has shown that three types of PIs were up-regulated when a *Microcystis* strain was grown on a phosphate-deplete medium and down-regulated when nitrogen was limited. These findings were later corroborated for another *Microcystis* strain [76]. Furthermore, a higher PI content because of P-limitation led to lower IC_50_ values of *Daphnia*’s inhibited proteases and vice versa if *Microcystis* was grown on an N-deplete medium; a lower PI content also led to higher somatic growth rates [34].

*Daphnia* show species-specific protease band patterns of gut extracts in activity-stained SDS-PAGE: In *D. pulex*, the band pattern consists of eight different bands that revealed either predominantly trypsin or chymotrypsin activity [58], whereas *D. magna* has a pattern with eight bands (five trypsins, four chymotrypsins; [77]). Those patterns differ between *D. magna* populations in height but not in the number of bands [78]. If *Daphnia*’s diet contains trypsin inhibitors, the trypsin bands become weaker; if *Daphnia*’s diet contains chymotrypsin inhibitors, it has been shown that within 24 h, the chymotrypsin band pattern shifts, with the lowest band dividing into two separate bands probably representing two different isoforms [60]. This shift in band pattern can similarly be provoked when pure PIs are ingested via liposomes, i.e., this shift into different chymotrypsin isoforms is probably due to PIs and not caused by any other cyanobacterial factors [77]. Furthermore, chymotrypsin activity increased and trypsin activity decreased when *D. magna* clones were fed with trypsin inhibitors; furthermore, the residual trypsin activities and IC_50_ values were positively correlated to the difference in tolerance of the different *D. magna* clones to the dietary trypsin inhibitors [8]. This demonstrates intraspecific differences in tolerance to PIs.

In addition, on the genetic level, *Daphnia* show target-specific responses to PIs. In transcriptome studies, it has been observed that the genes of digestive proteases were regulated depending on whether different *Daphnia* species had or had not ingested PI-producing cyanobacteria [79,80]. In *D. magna*, six different protease genes (three trypsins and three chymotrypsins) have clearly been identified to underlie the protease band pattern of active proteases in *D. magna*’s gut [60]. Those genes were up-regulated when *D. magna* was grown on PI-producing cyanobacteria [8,60,65]. This increase in gene expression proved to be adaptive: When *D. magna* mothers were fed with a trypsin-inhibitor-producing cyanobacterium, the increased protease gene expression was maternally transferred to their untreated offspring. In turn, the offspring showed a higher somatic growth rate on dietary trypsin inhibitors in comparison to the offspring from naïve mothers [81].

It has been shown that *Daphnia* populations can be locally adapted to PIs [82,83,84,85], which is due to the presence of PI-producing cyanobacteria in the lakes or ponds of origin in the past. Interestingly, two populations that have been shown to be tolerant to a cyanobacterium that produces different chymotrypsin inhibitors of the class of Ahp-cyclodepsipeptides are similarly tolerant to another cyano-bacterium that produces different chymotrypsin inhibitors of the same class [85]. Although those populations altogether were tolerant to both cyanobacteria, some of the single clones were tolerant to one but not the other strain. Interestingly, one of the tolerant populations consisted of several clones that showed a trade-off between somatic growth rate and egg number, demonstrating that different clones followed different strategies to cope with dietary chymotrypsin inhibitors. Furthermore, the molecular basis underlying local adaptation to PIs has been thoroughly investigated: Population tolerance has been linked to the production of different protease isoforms with higher IC_50_ values and positive selection with a subsequent reduction in the copy numbers of protease genes [83,84].

It has been shown that both sensitive and locally adapted populations show intra-population differences by containing both tolerant and sensitive clones [85]. Therefore, if a freshwater ecosystem becomes increasingly eutrophic due to sewage drain or over-fertilization, a sensitive *Daphnia* population has the potential to adapt to PI-producing cyanobacterial blooms by genotype frequency shifts.

## 4. Anatoxin-a (ATX)

The neurotoxic alkaloid ATX, also known as the Very Fast Death Factor [86], is an exceptionally dangerous toxin for humans, livestock and ecosystems [87] because it acts very quickly, and an antidote is unknown. It has already been detected in dietary supplements containing cyanobacteria [88]. In vertebrates, ATX binds irreversibly to nicotine-acetylcholine receptors with a higher affinity than acetylcholine and is not hydrolysed by acetylcholinesterase [89]. In humans, irreversible binding of ATX to nicotine-acetylcholine receptors can result in permanent stimulation of muscles, which in turn leads to paralysis and death due to respiratory arrest [90].

ATX occurs globally in natural lake blooms [26,27,39,87,91,92,93,94]. Interestingly, ATX can occur in increasing volumes as a single toxin in blooms and not only in mixtures with other cyanotoxins [39]. For example, in Lake Garda, *Planktotrix rubescens*, which is known to produce a variety of cyanotoxins (e.g., [35,65]), is currently substituted by the ATX-producer *Tychonema bourellyi* [39]. Besides *Tychonema* [94], different cyanobacterial species are known to produce ATX, e.g., *Anabaena* [26,95,96], *Pseudoanabaena*, *Planktothrix* [26] and *Aphanizomenon* [97]. The cyanobacteria are ingested by zooplankton; ATX is thus transferred through the food web and affects both fish and zooplankton [98].

In different *Daphnia* species, it has been shown that ATX negatively affects somatic and population growth rates, fecundity and survival [96,99,100]. Furthermore, Bownik and Pawlik-Skowrońska [101] observed sub-lethal effects of ATX: time- and concentration-dependent inhibition of *Daphnia*’s heart rate, alteration of oxygen consumption and induction of behavioural changes by this neurotoxin, i.e., abnormal circular swimming, altered swimming speed and reduced thoracic limb activity. Interestingly, ATX not only has a negative but also a positive effect on *D. dentifera* by preventing infection by the fungal parasite *Metschnikowia* [95]. Furthermore, *Daphnia* have been demonstrated to respond to ATX by up-regulating several nicotine-acetylcholine receptor genes; this response is adaptive since it is maternally transferred and results in higher growth rates of unexposed offspring [99]. It is unknown but worth investigating whether *Daphnia* can also respond to ATX by increasing the activity of detoxifying enzymes, such as peroxidases and glutathione S-transferases, as has been shown for aquatic plants [102].

## 5. Microcystins (MCY)

MCYs are the most studied toxins in *Daphnia*–cyanobacteria interactions. Many publications that describe toxic effects on *Daphnia* actually refer to MCY-producing cyanobacteria. In light of the plethora of cyanotoxins and their manifold effects on *Daphnia*, I propose to cease using the term “toxic cyanobacterium” when referring to MCY-producing cyanobacteria in future studies but to actually name the examined cyanotoxin.

MCYs belong to the most abundant cyanotoxins in European lakes and have been detected in greater frequency than CYN or ATX [39]. Although an exceptionally high number of MCYs are known, cyanobacterial blooms are mostly dominated by MCY-RR, -LR and -YR [103]. Those MCY-variants have been shown to negatively affect *Daphnia*’s food uptake, somatic and population growth rate, mortality, longevity and fecundity [9,65,104,105,106,107]. Furthermore, MCY-LR also reduced the beat rates of the thoracic legs, mandibles and second antennae as well as the activity of the foregut and stimulated the midgut muscles [108]. MCY-LR also affected *Daphnia*’s behaviour by inhibiting swimming speed and hopping frequency [109]; MCY-LF had the same effect. On a molecular level, MCY-LR has been shown to inhibit protein phosphatases I and IIa of *Daphnia* in vitro [110,111].

Despite these negative effects, it has been demonstrated that if *Daphnia* ingest MCY-producing cyanobacteria, their offspring show an increase in fitness in comparison to offspring from MCY-naïve mothers [112,113,114]. This means that the mothers must possess adaptive mechanisms that are then transferred to their offspring. Furthermore, not all individual *Daphnia* clones are susceptible to MCY [115], and intraspecific differences in tolerance have been observed both in experimental and natural *Daphnia* populations [116,117,118]. Furthermore, Lemaire et al. [115] found that cyanobacterial toxicity effects on *Daphnia* were dependent on both the MCY-producing cyanobacterial strain and the *Daphnia* clone. Interestingly, when *Daphnia* clones and cyanobacterial strains originated from the same pond, fewer toxic effects on *Daphnia* were observed [115], hinting at local adaptation.

Rapid microevolution of a *Daphnia* population to become locally adapted to MCY-LR has been observed by Hairston et al. [40]. In this study, *Daphnia* clones were resurrected from ephippia from different layers of lake sediment cores. Those different layers represented different periods in which the lake was either oligotrophic or became increasingly eutrophic. This eutrophication was accompanied by the frequent occurrence of (MCY-producing) cyanobacteria. Hairston et al. [40] could show that the *Daphnia* from eutrophic times were less affected when fed with an MCY-LR-producing *Microcystis* strain than the *Daphnia* from times when the lake had still been oligotrophic. Interestingly, the same *Daphnia* population has recently been shown to undergo reversed evolution [119]: The re-oligotrophication of the lake due to reduced nutrient input led to a decrease in (MCY-producing) cyanobacteria. Subsequently, the locally adapted *Daphnia* population returned to a state where the population’s clones were again highly sensitive to an MCY-producing *Microcystis* strain.

The underlying molecular basis of tolerance or sensitivity to MCY is still not finally clarified, and the major molecular response mechanism has not been identified. Several mechanisms probably underlie the tolerance or sensitivity of certain *Daphnia* clones to MCY. This is underpinned by the finding that in both transcriptome/microarray [79,80,120] and proteome studies [121,122], several different (metabolic) pathways were affected when *Daphnia* were fed with MCY(-LR)-producing cyanobacteria.

Pflugmacher et al. [110] proposed that, as the first step in the detoxification of MCY-LR in *Daphnia*, the detoxifying enzyme glutathione S-transferase (GST) forms a glutathione-microcystin conjugate. However, none or only a few GST genes were found to be up-regulated in response to dietary MCY-LR in transcriptome and microarray studies of two different *Daphnia* species [79,80]. Short-term exposure to an MCY-producing cyanobacterium increased GST gene expression, whereas long-term exposure decreased expression of the same gene [123]. Similarly, GST activity decreased during long-term exposure to a high dosage of MCY-LR [111]. In contrast, a higher GST and malate dehydrogenase activity was observed in response to MCY in *D. carinata*, which was maternally transferred to the offspring generation [124]. Such a maternal transfer is cost-intensive and should therefore be adaptive. However, the role of GST has been called into question by Sadler and Von Elert [125], because in their study neither glutathione-conjugates of MCY-LR nor a decrease in unconjugated MCY-LR was detected in *D. magna*. It might be that the GST in *D. magna* is involved in the oxidative stress response rather than in the specific detoxification of MCYs.

Further general molecular responses have been demonstrated to be involved in developing the tolerance to dietary MCY(-LR) in *Daphnia*: e.g., the regulation of certain transporters probably used to export microcystins from the cells or to prevent MCY import [65,80,125], up-regulation of different oxidative stress genes [79] and the involvement of epigenetics (i.e., differential methylation of serine and threonine amino acids [126]). Furthermore, Drugă et al. [116] and Schwarzenberger et al. [127] observed that a ubiquitin-conjugating enzyme (UBQ) gene was up-regulated in the presence of MCY(-LR)-producing cyanobacteria, which points towards the possibility that MCY might be degraded by UBQ.

But *Daphnia*’s own genetic fixture nor its ability to phenotypically respond is not solely responsible for the tolerance to MCY: The gut microbiomes of susceptible and tolerant *Daphnia* clones differ markedly, and Macke et al. [128] could show that the microbiome of a tolerant clone transplanted into a germ-free recipient clone resulted in increased tolerance to an MCY-producing cyanobacterium.

Since Macke et al. [129] found that there is also a strong interaction between the host genotype, its microbiome and its surrounding free-living microbial community, it is likely that a shift in the cyanobacterial composition of a freshwater system can lead to a shift in *Daphnia*’s microbiome composition. Therefore, it is highly likely that the microbiome also plays a major role in tolerance to other cyanotoxins besides MCY.

## 6. Control of Toxic Cyanobacterial Blooms by *Daphnia*?

This review summarizes that *Daphnia* are not helpless victims of dietary cyanotoxins but have the ability to respond and adapt to different cyanotoxins. Some underlying mechanisms can generally be observed in response to different cyanotoxins (e.g., GST and transporters (cf. [65])), while others are highly specific (e.g., serine proteases and nicotine acetylcholine receptors). However, the more cyanotoxins are present in a single food alga or in a food source that consists of a mixture of cyanobacteria that produces several cyanotoxins, the effect on *Daphnia* should be graver or even fatal. For example, the MCY-producing, wild-type of *Microcystis aeruginosa* PCC7806 that also produces PIs [75] affects survival, age at reproduction and neonate size of different *Daphnia* species more strongly than its MCY-free, knock-out mutant [104,107,130]. Furthermore, different *Planktothrix* strains differ in their impact on *Daphnia* putatively because of their different cyanotoxin composition [65].

For a long time, *Daphnia*’s capability to down-regulate cyanobacterial blooms is controversially discussed, and it is not finally clarified which are the decisive factors leading to the down-regulation of a bloom or the death of *Daphnia* populations (cf. [11,12,131,132,133]). Besides geographic and hydrologic factors [134], the presence of a specific cyanotoxin [14] and initial conditions before bloom formation [132,135,136], also the cyanotoxin composition of the bloom-forming cyanobacteria might be a pivotal factor.

## 7. Outlook: *Daphnia*–Cyanobacteria Interactions in the Anthropocene

**Figure 1:**
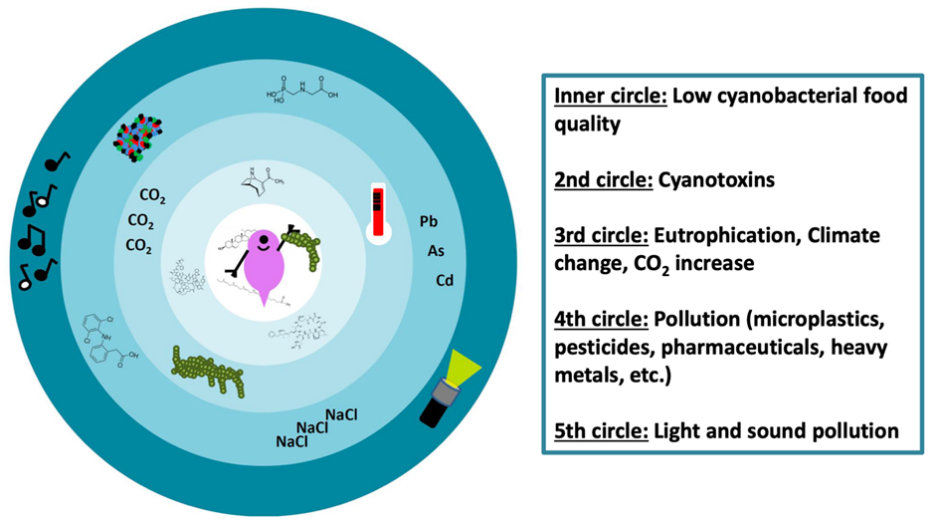
The “Circles of Hell” for Daphnia in the Anthropocene. The first two inner circles represent the effects of cyanobacteria on Daphnia with their additional constraints, i.e., low food quality plus cyanotoxicity. The third circle shows the factors that increase the frequency of cyanobacterial blooms or the strength of cyanotoxicity. The fourth and fifth circle show stressors that (might) have an additional effect on Daphnia ingesting cyanobacteria and their toxins (e.g., heavy metals such as Pb, As and Cd, road salts (NaCl) and light pollution).

The Anthropocene keeps ready a plethora of new and fast-increasing threats that will most likely further affect *Daphnia*–cyanotoxin interaction (Figure 1).

### 7.1. Anthropogenic Temperature Increase

There is, on the one hand, climate change and global warming: It has already been shown that protease gene expression is influenced by different temperatures in *Daphnia* [58] and that cyanobacterial toxicity increases in higher temperatures while the activity of proteases of *Daphnia* decreases [58]. In addition, in the case of an anatoxin-a-producing cyanobacterium, the high temperature was responsible for a further decrease in the population growth rate of *D. pulex* [96]. Akbar et al. [137] found that under heat stress, *Daphnia*’s fitness and gut microbiome was reduced when treated with microcystin-producing cyanobacteria. Interestingly, northern European (and probably colder) areas exhibited higher toxin diversity than Mediterranean areas [39]. Furthermore, a heat wave in 2015 also led to a higher cyanobacterial biomass to the north of selected (heat-tolerant) genotypes [39]. Higher toxicity coupled with a higher biomass of cyanobacteria on a permanent level might render it impossible for *Daphnia* to control cyanobacterial blooms in the future.

### 7.2. Anthropogenic Light and Sound Pollution

Besides climatic changes, further abiotic anthropogenic stressors have the potential to disrupt the general (the “normal”) *Daphnia*–cyanobacteria interaction. It has already been demonstrated that artificial light at night/light pollution changes the expression of *Daphnia* protease genes and that increased protease activity leads to better control of a protease inhibitor-producing cyanobacterium [56]. Furthermore, increasing sound pollution might be problematic for the control of cyanobacterial blooms: A mesocosm study by Lürling and Tolman [138] showed that ultrasound strongly suppressed *Daphnia*, thus freeing phytoplankton from grazing control.

### 7.3. Anthropogenic CO_2_ Increase

The burning of fossil feedstock leads to a heavy rise in atmospheric CO_2_. In the ocean, the alga *Karlodinium veneficum* produced higher levels of karlotoxin due to increasing CO_2_ (especially in P-limited cultures; [139]). The authors concluded that over the coming decades, rising CO_2_ levels could substantially increase karlotoxin damage to food webs in the often P-limited estuaries in which *Karlodinium* blooms occur. Such a scenario under increasing CO_2_ levels is also imaginable for freshwater ecosystems in which *Daphnia* occur. In addition, in cyanobacteria, P-limitation results in a higher production of protease inhibitors, which is accompanied by lower IC_50_ values for *D. magna* gut proteases [34]. Furthermore, rising CO_2_ and global warming favour cyanobacterial blooms by stimulating their N_2_-fixation activity [140].

### 7.4. Anthropogenic Pollutants

The increasing production and especially the degradation of plastics in the environment might pose another threat to freshwater organisms. Several studies dealt with the effects of both microplastics (e.g., [141,142,143]) and temperature on *Daphnia* [144,145]. It might well be that a potentially deleterious effect of microplastic also disrupts the interaction of this grazer with cyanobacteria, especially in light of climate change: This might lead to a higher susceptibility of *Daphnia* to cyanotoxins.

Besides microplastics, road salts are also washed into freshwater ecosystems, and it has been shown that increasing chloride concentrations led to a decline in the abundance, biomass, and richness of zooplankton [146]. The addition of fatty acids and sterols both influence *Daphnia*’s tolerance to salinization [147]. Since cyanobacteria lack both types of those fats, a salinization effect on *Daphnia*-cyanobacteria can be expected. Furthermore, road salts also disrupt *Daphnia*’s circadian clock gene expression [148], which might influence *Daphnia*’s ability to cope with cyanobacterial protease inhibitors since the gene expression of gut proteases is regulated by the circadian clock [56].

Anthropogenic chemicals (i.e., pharmaceuticals, herbicides, insecticides, heavy metals, etc.) are continuously discharged into the environment leading to a chronic exposure of organisms to single or combined toxic compounds. It has been shown that *Daphnia* that were exposed to one or more stressors in multigenerational approaches, either in combination or successively, showed lower reproduction [149], lower resistance to an additional toxin (i.e., glyphosate) and starvation [150]. Since glyphosate and cyanobacterial anatoxin-a both attack nicotine acetylcholine receptors of arthropods [151], a combined exposure with anthropogenic chemicals and natural toxins might further reduce *Daphnia*’s ability to cope with cyanobacterial blooms.

### 7.5. Combined (Natural and/or Anthropogenic) Stressors

Initial studies dealt with the effect of combined stressors (natural and/or anthropogenic) on *Daphnia*, e.g., salt and the antiozonant 6PPD [152], microplastic and temperature [144], lithium and light stress [153], microcystin and temperature [154] and a *Daphnia* transcriptome study across 12 different environmental stressors [155]. However, most studies deal with the effect of combined stressors on a single organism and not on species interaction. Since *Daphnia* genotype-dependent responses to single or mixed stressors have been found [156], clonal differences need to be taken into account in future studies on stressor/cyanotoxin effects on *Daphnia*–cyanobacteria interactions. Furthermore, the combination of only two stressors was mostly investigated in former studies, which unfortunately does not reflect the current situation of an increasing number of anthropogenic stressors in freshwater ecosystems.

## 8. Conclusions

It is mandatory that in future risk assessments of cyanotoxins, several anthropogenic stressors need to be taken into account. Since cyanobacteria are or will be used in diverse applications (e.g., biofuel and dietary supplements) it is important to monitor which influences suppress or increase the production of cyanotoxins. This means not only dealing with the well-investigated microcystins, but also considering all kinds of cyanotoxins that affect human health and the environment (cf. [157]). Furthermore, screening for different cyanotoxins is necessary when monitoring cyanobacterial blooms.

Right now, the future of clean (i.e., cyanotoxin-free) drinking water is endangered by a multitude of anthropogenic stressors. Therefore, fast action is needed for reducing the number and concentration of pollutants, i.e., by banning certain anthropogenic chemicals, establishing smart light concepts, reducing sound pollution, establishing working recycling systems to prevent plastic degradation in the environment, lowering CO_2_ production/investing in technologies for direct CO_2_ capture, etc. Otherwise, the control of harmful cyanobacterial blooms due to physiological adaptations of *Daphnia* genotypes to cyanotoxins and the occurrence of locally adapted *Daphnia* populations might become impossible.

## Figures and Tables

**Table 1:** Effects of the five different toxin types on Daphnia and physiological and (micro)evolutionary adaptations (with numbers of citations in brackets) of Daphnia in response to the different toxins. Empty fields represent lack of evidence or missing data. Different enzymes can both be affected by cyanotoxins or be involved in adaptation. UBQ = ubiquitin conjugating enzyme; PPI and IIa = protein phosphatases I and IIa; CXP = carboxypeptidase; GST = glutathione-S-transferase; NAR = nicotine-acetylcholine receptors).

		Toxin Effects									Adaptations of *Daphnia*				
		Mortality	Growth	Fecundity	Ingestion	Oxygen Consumption	Heart Rate	Behaviour	Uptake in Tissues	Enzymes		Microbiome	Genes	Maternal Effects	Local Adaptation
**Toxin types**	**Cylindro-** **spermopsin**	x	x						x		x (changes in GST activity (51))				
	**Carboxypeptidase** **inhibitors**									x (Inhibition of CXP)			x (changes in CXP expression (65))		
	**Protease** **inhibitors**	x	x	x	x					x (inhibition of proteases)	x (change in protease activity & band patterns (60, 77); protease isoforms with higher IC_50_ values (8))		x (changes in gene expression (8, 60, 65, 79, 80); copy number variation (84); positive selection (83))	x (81)	x (82–85)
	**Microcystins**	x	x	x	x			x		x (inhibition of PPI and IIa)	x (effect on GST (124) and malate dehydrogenase (124))	x (128)	x (expression changes in molecular pathways (79, 80, 120-122); epigenetic effects (126) change in GST (123), UBQ (127), oxidative stress (79) and transporter gene expression (65, 80, 125))	x (112–114)	x (40, 119)
	**Anatoxin-a**	x	x	x		x	x	x					x (change in NAR gene expression (99))	x (99)	

## Data Availability

Not applicable.
